# CORRECT-Net: A Multimodal Vibration–Current Fusion Network for Coal–Rock Cutting State Recognition in Shearers

**DOI:** 10.3390/s26165181

**Published:** 2026-08-16

**Authors:** Lijuan Zhao, Zhanpeng Zhang, Yadong Wang, Tiangu Wu, Jie Hao

**Affiliations:** 1 School of Mechanical Engineering, Liaoning Technical University, Fuxin 123000, China; 2 Liaoning Province Key Laboratory of Large-Scale Industrial and Mining Equipment, Fuxin 123000, China

**Keywords:** shearer, coal–rock cutting state recognition, multimodal fusion, CORRECT-Net, transfer learning

## Abstract

Coal–rock cutting state recognition is essential for adaptive cutting and intelligent speed regulation in shearers. To address the limited representational capability of individual signals, the confusion between adjacent gangue-bearing cutting conditions, and the domain discrepancy between simulation and experimental data, a vibration–current multimodal fusion method based on CORRECT-Net is proposed. First, an EDEM–RecurDyn–MATLAB/Simulink co-simulation system was developed to generate cutting records for four coal–rock states. After screening for physical equivalence and label conflicts, 158 valid records were retained and grouped into 150 physical-condition groups, which were partitioned at the group level into training, validation, and test sets. Subsequently, SincNet was employed to extract frequency-band-constrained features, a Transformer was used to model long-range temporal dependencies, and a residual importance-guided GATv2 module was introduced to perform cross-modal fusion of vibration-impact and current-load features. On 2500 test windows, CORRECT-Net achieved an accuracy of 96.20% ± 0.11%, a macro-F1 score of 95.14% ± 0.21%, and a hazardous-condition miss rate of 0.58% ± 0.13%. Compared with the multimodal 1D-CNN, TCN, and Bi-LSTM models, CORRECT-Net improved the accuracy by 8.80, 4.00, and 2.08 percentage points, respectively. In the progressive ablation study, the accuracy increased from 87.40% ± 0.26% to 96.20% ± 0.11%, while the macro-F1 score increased from 84.57% ± 0.34% to 95.14% ± 0.21%. Under Gaussian noise with a standard deviation of 0.05, the model retained an accuracy of 92.76% ± 0.24%. When the vibration and current modalities were separately unavailable, the corresponding accuracies were 86.56% ± 0.37% and 92.44% ± 0.25%, respectively. A five-fold simulation-to-experiment transfer evaluation was further conducted at the independent-run level using five experimental records per class. Without adaptation using experimental samples, the model achieved an accuracy of 91.33% ± 5.19%. When 20% and 50% of the experimental windows were used for adaptation, the accuracy increased to 96.33% ± 0.75% and 98.67% ± 1.39%, respectively. These results demonstrate that CORRECT-Net effectively integrates mechanical vibration responses and motor-load information and, under the present simulation and experimental conditions, achieves high recognition accuracy, a low hazardous-condition miss rate, and effective adaptability to the experimental domain.

## 1. Introduction

Recognition of coal–rock cutting states is essential for adaptive cutting, intelligent speed regulation, and unmanned longwall mining with shearers, and it plays an important role in improving the safety and intelligence of mining operations under complex geological conditions [1,2]. Existing coal–rock recognition methods mainly include near-infrared and spectroscopic sensing, infrared thermography, machine vision, acoustic and vibration monitoring, and multisensor fusion [3,4,5,6,7,8,9,10]. Regarding spectroscopic recognition, Lv et al. developed a near-infrared spectral dataset containing multiple types of coal and coal-bearing rock, providing fundamental data for training coal–rock recognition models [3]. Yang et al. combined spectral dimensional transformation with CBAM-ViT for coal–rock recognition, demonstrating the effectiveness of deep attention models in extracting spectral features [4]. Xu et al. integrated laser-induced breakdown spectroscopy with deep learning for coal–gangue classification, showing that deep spectral features can reflect material differences between coal and gangue [5]. In image-based recognition, Sun et al. proposed a deep-learning-based coal–rock image recognition method for complex underground environments [6]. Zhao et al. employed infrared thermal images and an improved U-Net to identify anomalous regions associated with coal–rock failure [7], while Qiao et al. developed a multimodule fusion network for coal–rock interface recognition [8]. In addition, Liu et al. fused laser point-cloud and image information for coal–rock recognition at the mining face. Wang et al. further incorporated differences in coal–rock hardness and used multisensor information fusion to achieve dynamic coal–rock interface recognition, indicating that multisource information can enhance perception under complex operating conditions [9,10].

Despite these advances, several challenges remain in recognizing coal–rock cutting states under complex conditions. Image-, infrared-, and spectrum-based methods are susceptible to dust, water mist, illumination variations, and occlusion. A single vibration or current signal is insufficient to simultaneously characterize gangue impacts, coal–rock fragmentation, and variations in cutting load. Moreover, experimental cutting data are costly to acquire and difficult to reproduce under consistent operating conditions. Distribution discrepancies between simulation and experimental data may further limit the cross-domain adaptability of recognition models [11,12]. Therefore, a key unresolved problem is how to jointly exploit complementary vibration-impact and motor-load information while maintaining reliable recognition when the model is transferred from simulation data to experimentally acquired signals.

To address the limited representational capability of individual modalities, the confusion between adjacent gangue-bearing cutting states, and the distribution discrepancy between simulation and experimental data, this study proposes a vibration–current multimodal fusion method based on CORRECT-Net. Physically equivalent simulation records are grouped according to their effective parameters, and the physical-condition groups are partitioned before time-window generation. This strategy prevents physically equivalent records and their derived windows from being distributed across different subsets, thereby reducing the risk of information leakage. SincNet is employed to adaptively extract frequency-band-related features, while a Transformer is used to model long-range temporal dependencies. A residual importance-guided GATv2 module is then introduced to establish cross-modal associations between vibration-impact information and current-load information, thereby improving the discrimination of adjacent coal–rock cutting states. Finally, an independent-run-based five-fold transfer evaluation is conducted to assess the transfer of the model from simulation data to experimental data and its adaptability to the experimental domain.

## 2. Development of the Coal–Rock Cutting System Model

To address the high cost of acquiring experimental data and the limited coverage of coal–rock cutting conditions, a coupled discrete-element and multibody-dynamics model was developed. The model comprises a discrete-element model of the coal–rock mass, a dynamic model of the shearer cutting unit, and a cutting-motor model. It was used to obtain the cutting-drum vibration response and motor-current signal under different coal–rock structural conditions. Previous studies have demonstrated that EDEM–RecurDyn co-simulation can characterize coal–rock fragmentation, cutting loads, and the dynamic responses of mechanical systems, thereby providing a suitable modeling framework for generating cutting-state data under complex coal–rock conditions [13].

### 2.1. Development of the Shearer Cutting Unit Model

**Figure 1:**
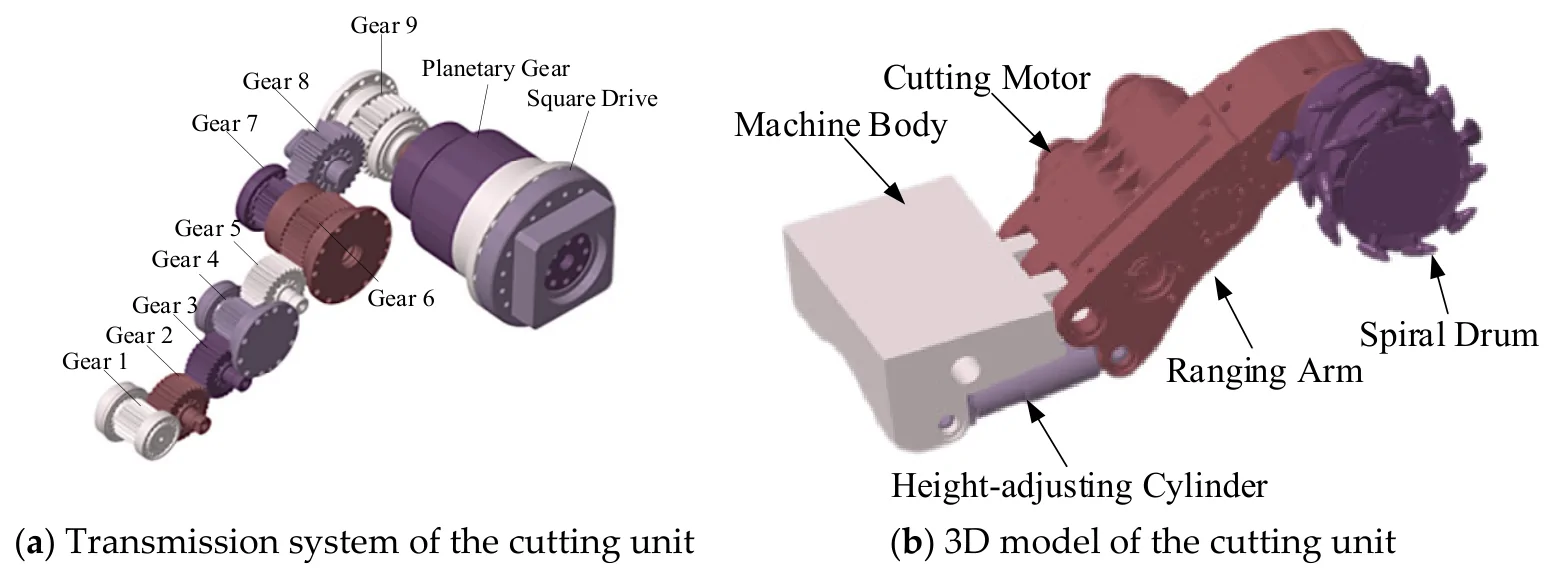
Three-dimensional model of the shearer cutting unit and transmission system.

An MG2 × 55/250-BWD thin-seam shearer was selected as the research object. A three-dimensional model of the cutting unit, including the cutting drum, ranging arm, and multistage gear transmission system, was developed using Creo Parametric 8, as shown in Figure 1.

To represent the contact deformation and impact generated during gear meshing, a nonlinear contact model was established for each gear pair [14]. The normal contact force is expressed as

(1) $$F_{n}=\left\{\begin{array}{cc} K\delta^{p}+Cv_{n} & \delta >0\\ 0 & \delta \leq 0 \end{array}\right.$$

where *F_n_* is the normal contact force; *δ* is the penetration depth, mm; *K* is the contact stiffness, N/mm; *C* is the contact damping coefficient, N·s/mm; *v_n_* is the normal relative velocity at the contact point, mm/s; and *p* is the nonlinear exponent.

The penetration depth between gears *i* and *j* is calculated from the relative positions of the two contacting bodies as

(2) $$\delta^{ij}=r_{t}+r_{p}-\sqrt{({\bar{u}}_{x}^{ij}{)}^{2}+({\bar{u}}_{y}^{ij}{)}^{2}}$$

where *δ^ij^* is the penetration depth between gears *i* and *j*; *r_t_* and *r_p_* are the equivalent contact-arc radii of the two contacting bodies at the contact point, respectively; and ${\bar{u}}_{x}^{ij}$ and ${\bar{u}}_{y}^{ij}$ are the X- and Y-components of the relative-position vector between the two arc centers in the local coordinate system, respectively.

The contact parameters assigned to the gear pairs are listed in Table 1. The maximum penetration depth defines the penetration range over which the contact damping term is applied.

**Figure 2:**
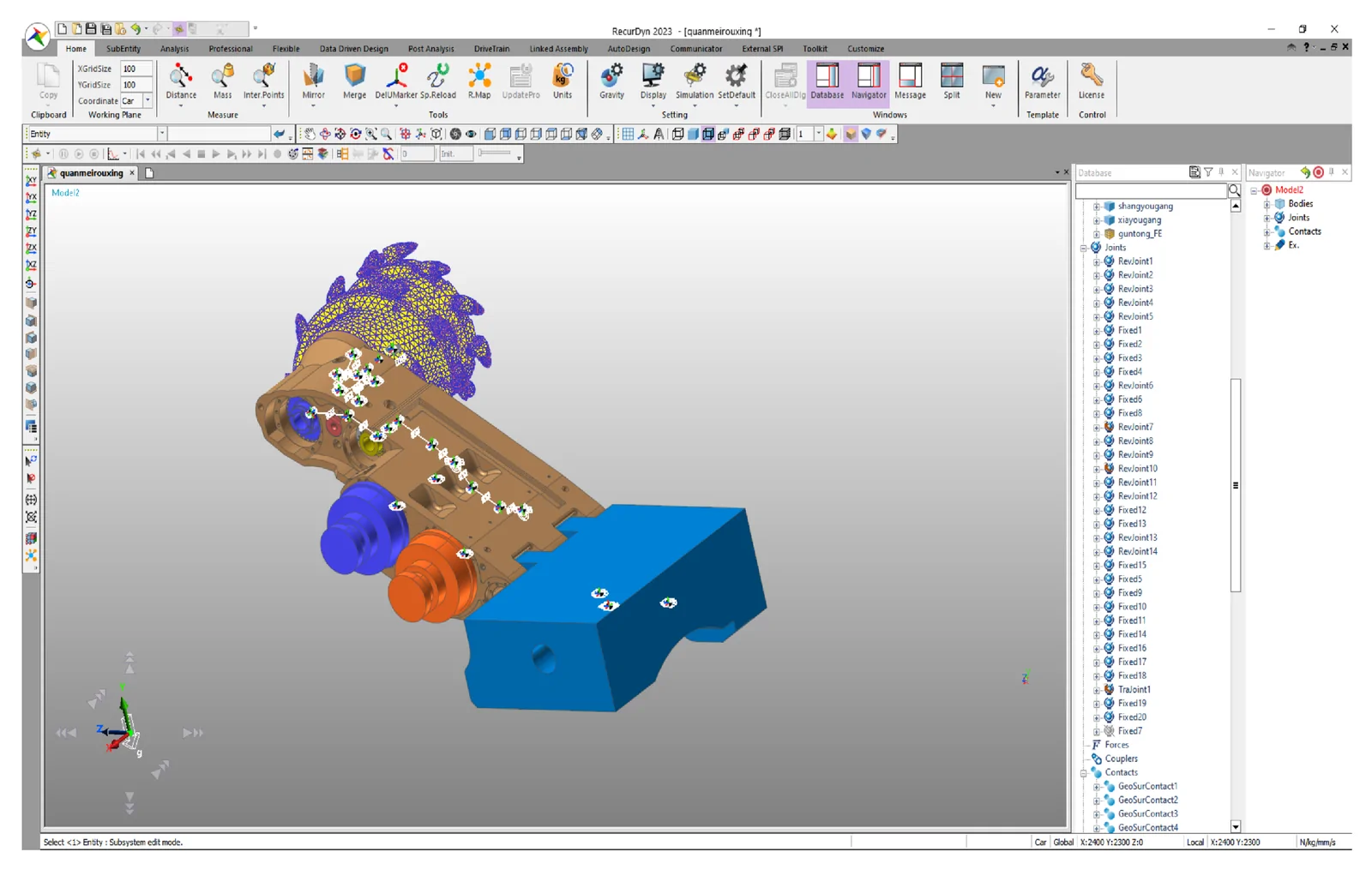
Rigid–flexible coupled model of the shearer cutting unit.

Because the helical cutting drum is subjected to substantial impact loads during cutting, it was modeled as a flexible body, whereas the remaining components were treated as rigid bodies. The resulting rigid–flexible coupled model of the shearer cutting unit is shown in Figure 2.

### 2.2. Development of the Coal–Rock Discrete-Element Model

To develop the coal–rock discrete-element model, physical and mechanical tests were conducted on coal and gangue samples collected from Coal Seam No. 17 of the Yangcun Coal Mine in the Yanzhou mining area. Macroscopic properties, including density, elastic modulus, tensile strength, compressive strength, and Poisson’s ratio, were obtained, as listed in Table 2. Based on these measured properties, the particle contact, friction, and bonding parameters were determined through combined experimental and numerical calibration and were subsequently used to develop the coal–rock discrete-element model in EDEM. Previous studies have shown that combined experimental and numerical calibration can improve the capability of discrete-element models to represent coal–rock fragmentation responses during cutting [15].

**Figure 3:**
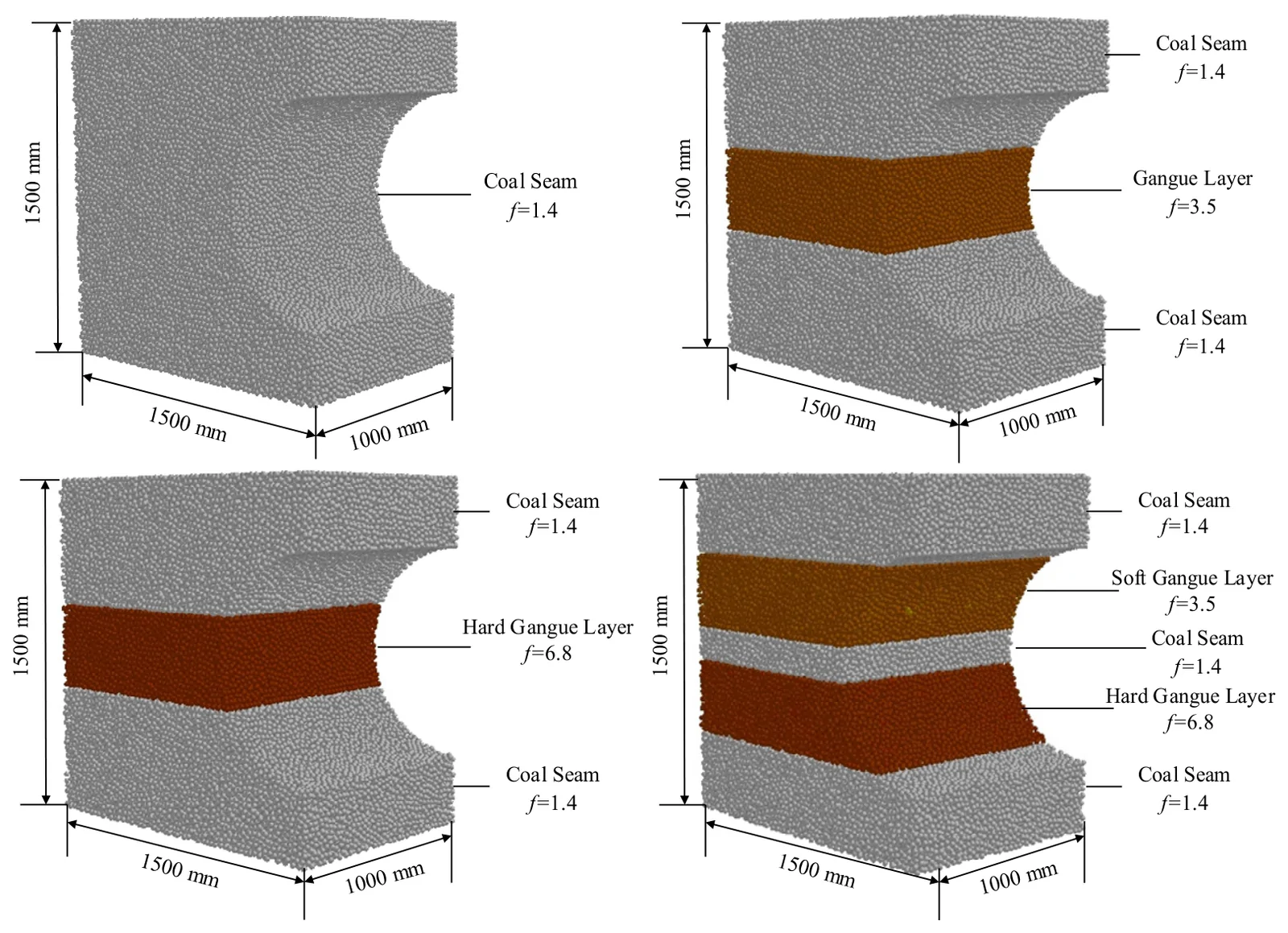
Representative discrete-element models of the coal wall.

Different coal–rock cutting conditions were constructed by varying the coal firmness coefficient, gangue firmness coefficient, and coal–rock volume ratio. After verifying the validity of the physical parameters and the consistency of the class labels, 158 valid simulation records were retained. Based on the parameters that were actually effective in the simulations, physically equivalent records were grouped into 150 physical-condition groups. These groups represented four cutting states: all-coal, a single gangue layer, double gangue layers, and all-rock. Their detailed composition is presented in Table 3. Representative discrete-element models of the four coal-wall conditions are shown in Figure 3.

### 2.3. Development of the Cutting-Motor Model

To obtain the current responses under different cutting loads, a three-phase induction-motor model was developed in MATLAB/Simulink using the synchronously rotating d–q reference frame. The Clarke–Park transformation was applied to convert the variables from the three-phase stationary reference frame to the two-axis rotating reference frame. The d–q-axis voltage equations are expressed as

(3) $$\left\{\begin{array}{l} U_{sd}=R_{s}I_{sd}+\frac{d}{dt}\psi_{sd}-\omega_{s}\psi_{sq}\\ U_{sq}=R_{s}I_{sq}+\frac{d}{dt}\psi_{sq}+\omega_{s}\psi_{sd}\\ U_{\mathrm{rd}}=R_{r}I_{rd}+\frac{d}{dt}\psi_{rd}-\left(\omega_{s}-N_{p}\omega_{r}\right)\psi_{rq}=0\\ U_{rq}=R_{r}I_{rq}+\frac{d}{dt}\psi_{rq}+\left(\omega_{s}-N_{p}\omega_{r}\right)\psi_{\mathrm{rd}}=0 \end{array}\right.$$

(4) $$T_{e}\;=\frac{2}{3}\;N_{p}\;\;\;(\psi_{sd}\;I_{sq\;}-\psi_{sq}\;I_{sd}\;)$$

where *U*, *I*, and *ψ* denote voltage, current, and flux linkage, respectively; *R* denotes resistance; the subscripts *s* and *r* denote stator and rotor quantities, respectively; the subscripts *d* and *q* denote the components along the d- and q-axes, respectively; *ω_s_* is the synchronous electrical angular velocity; *ω_r_* is the rotor mechanical angular velocity; and *N_p_* is the number of pole pairs. For the squirrel-cage rotor, the rotor voltages *U_rd_* and *U_rq_* are both zero; *T_e_* denotes the electromagnetic torque.

**Figure 4:**
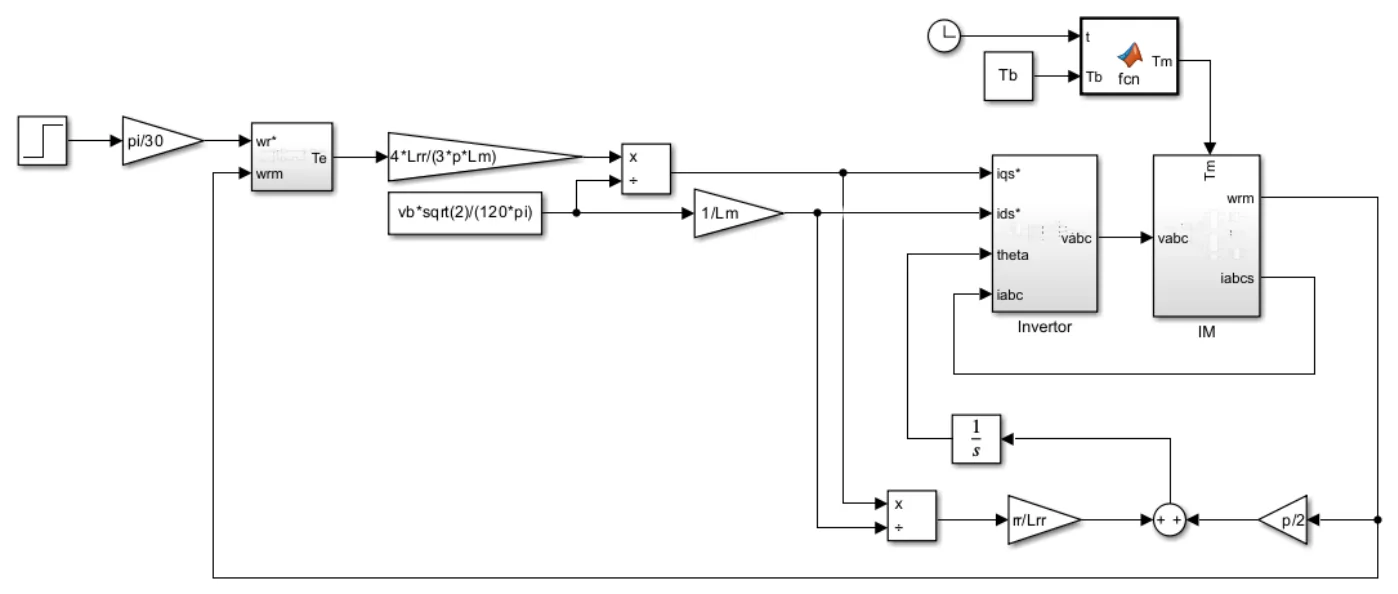
Simulink model of the cutting-motor drive system.

Using the parameters of a YBCS-55 mining three-phase induction motor, a cutting-motor drive model comprising a space-vector pulse-width modulation (SVPWM) inverter, a vector controller, and an induction-motor module was developed in MATLAB/Simulink R2022b. The main motor parameters are listed in Table 4, and the model structure is shown in Figure 4.

## 3. Acquisition of Coal–Rock Cutting-State Data

### 3.1. Development of the Coupled Coal–Rock Cutting Model

**Figure 5:**
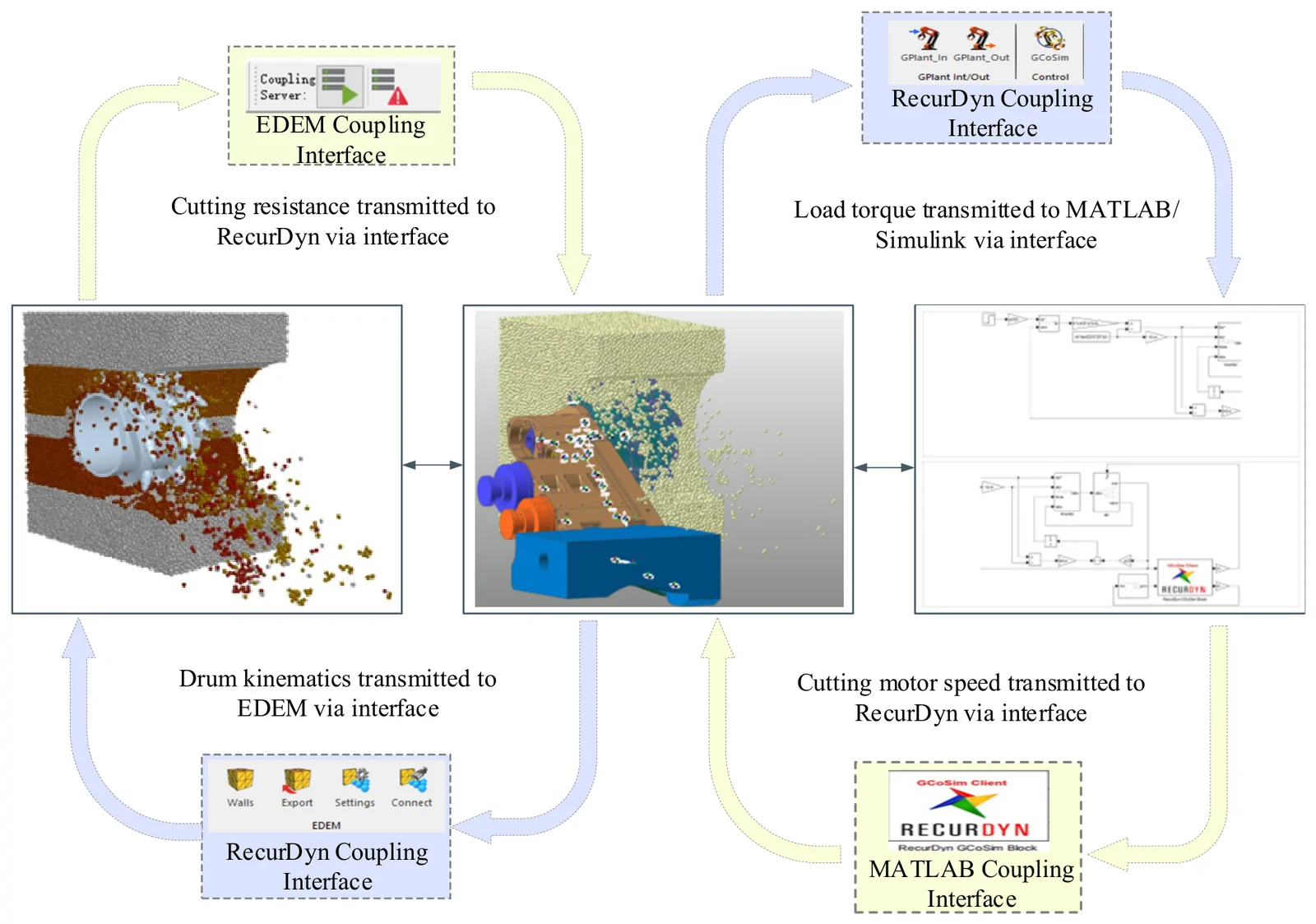
Workflow of the coal–rock cutting co-simulation system.

A coal-rock cutting co-simulation system was developed using the coupling interfaces among EDEM 2020, RecurDyn 2023, and MATLAB/Simulink R2022b. The overall workflow is illustrated in Figure 5. During the first 1 s of simulation, the system undergoes startup and position adjustment, while MATLAB/Simulink drives the cutting unit to accelerate to the prescribed rotational speed. After the cutting stage begins, RecurDyn transfers the motion parameters of the cutting drum to EDEM. EDEM then calculates the coal–rock cutting resistance and feeds it back to the dynamic model of the cutting unit. Meanwhile, RecurDyn transfers the drum load torque to MATLAB/Simulink, where the motor model calculates the corresponding rotational-speed and current responses. The drum vibration and cutting-motor current signals are exported using a common simulation time base to ensure temporal alignment between the two modalities. The total simulation duration is 10 s, the haulage speed is set to 4 m/min, and the cutting-drum rotational speed is fixed at 90 r/min.

### 3.2. Analysis of Simulation Results

To compare the vibration responses along different directions, the representative single hard-gangue-layer condition shown in Figure 3 was selected for analysis. Under this condition, the coal firmness coefficient was *f* = 2.38, the gangue firmness coefficient was *f* = 6.8, and the coal-to-gangue volume ratio was 3:1. The statistical results for the cutting-drum vibration acceleration along the three coordinate axes are presented in Table 5.

**Figure 6:**
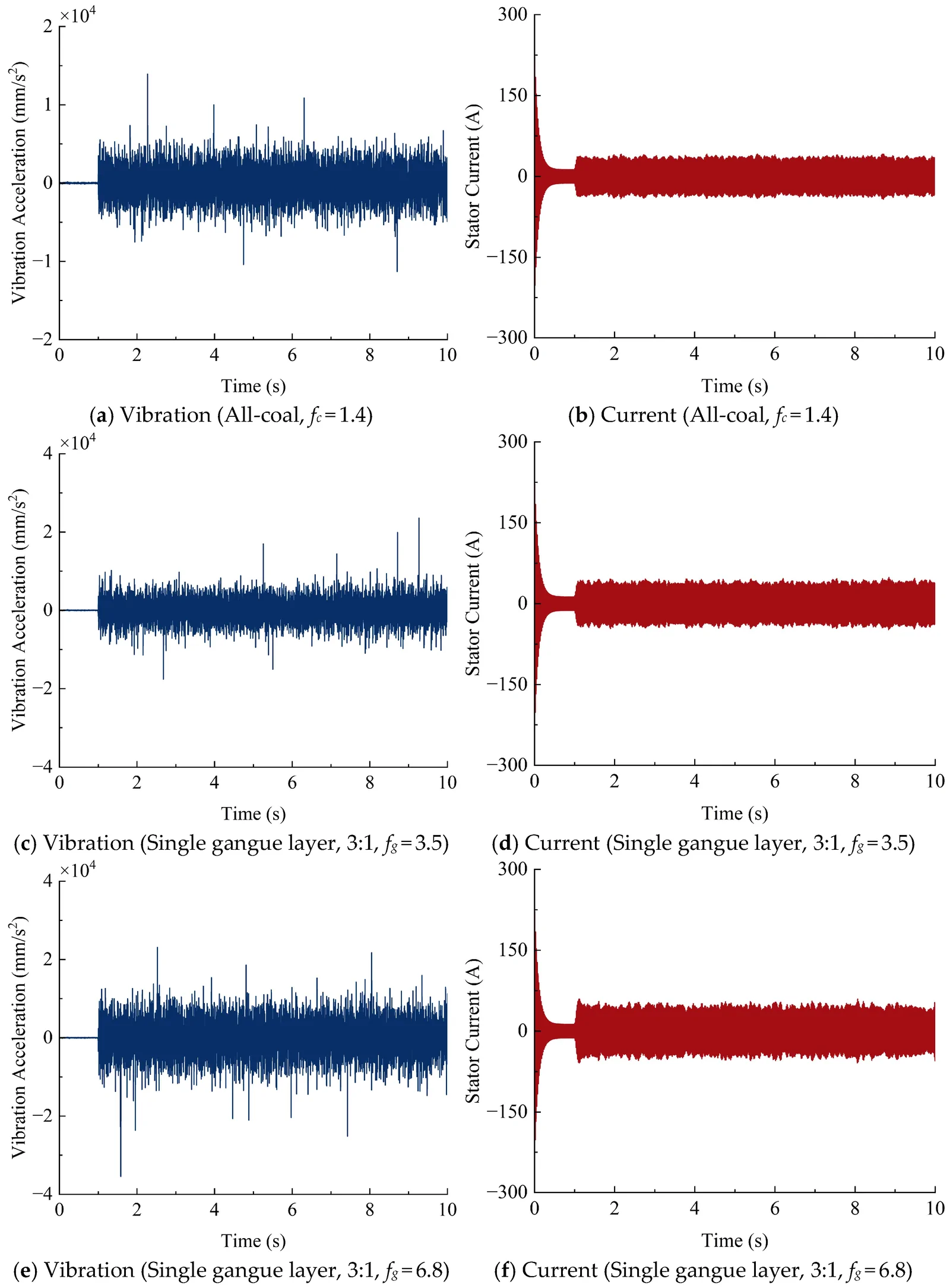

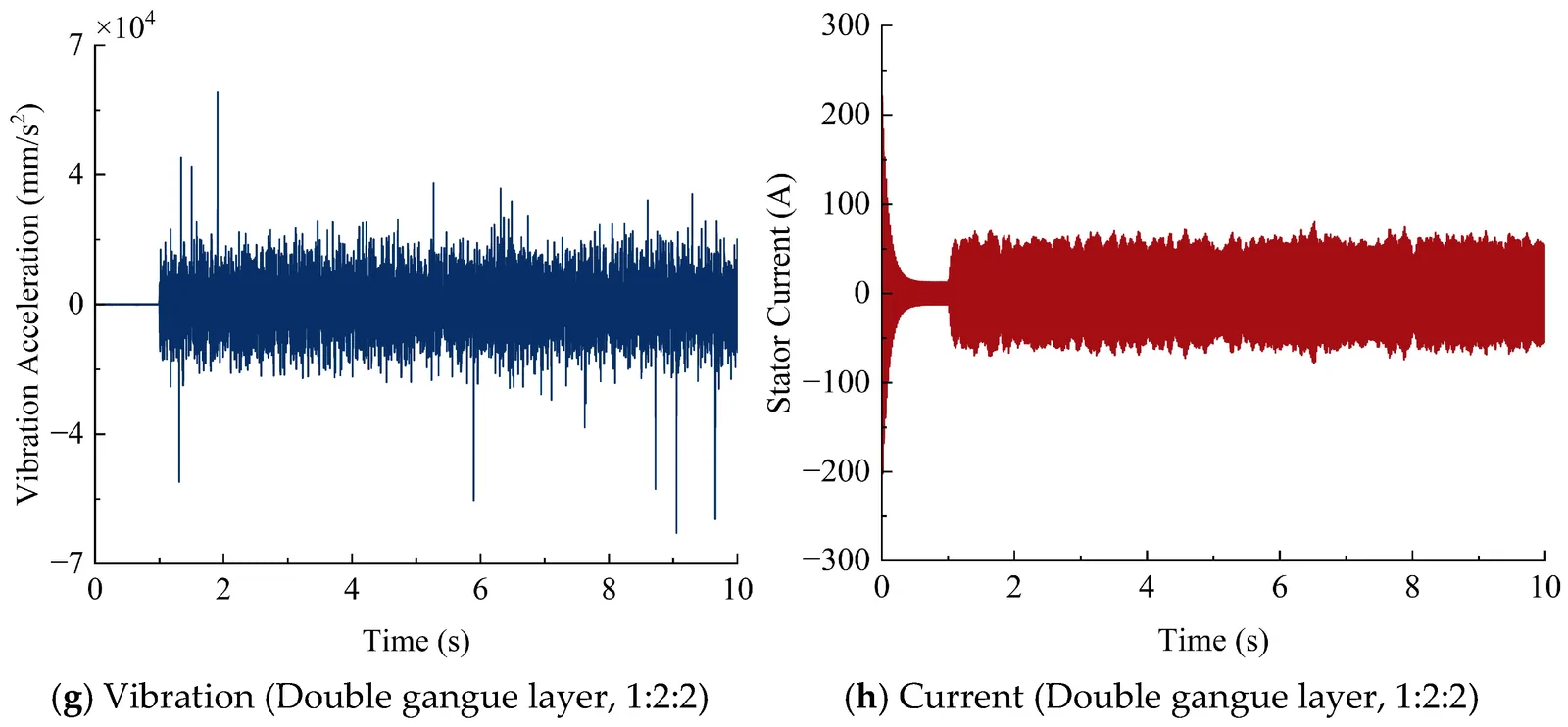
Vibration signals and current signals under different operating conditions.

As shown in Table 5, the RMS vibration acceleration in the Y-direction was 4384.0 mm·s^−2^, which was higher than those in the X- and Z-directions, at 1973.3 and 3287.5 mm·s^−2^, respectively. According to the coordinate definition of the model, the Y-direction corresponds to the primary cutting-load direction of the drum. Therefore, the Y-direction vibration acceleration was selected as the input of the vibration modality. The Y-direction vibration signals and cutting-motor current signals under representative cutting conditions are shown in Figure 6.

As illustrated in Figure 6, the vibration signals under different coal–rock cutting states exhibit distinct fluctuation patterns and local impact characteristics. The cutting-motor current signals also show different variations in response to changes in the cutting load. The vibration signal primarily reflects the mechanical responses induced by coal–rock fragmentation and gangue impacts, whereas the current signal characterizes variations in the cutting load and motor torque demand. Therefore, the two modalities provide complementary information for cutting-state recognition.

The vibration and current signals were synchronously exported using a common simulation time base. Each simulation record had a duration of 10 s and contained 10,000 sampling points, corresponding to a sampling frequency of approximately 1000 Hz. The first 1000 sampling points, corresponding to the approximately 1 s startup transient, were removed. This segment contains the acceleration, position-adjustment, and motor-current build-up responses before the cutting system reaches a stable operating state; its exclusion prevents non-cutting transient components from entering the window samples. The remaining 9000 points were retained as the steady-cutting segment. Within this segment, 100 window-starting positions were selected at equal intervals. Each window contained 1600 points, corresponding to approximately 1.6 s, and the average interval between adjacent starting positions was approximately 75 points. At the sampling frequency of 1000 Hz, the 1600-point window provides a frequency resolution of 0.625 Hz. With the cutting-drum rotational speed fixed at 90 r/min (1.5 r/s), each 1.6 s window covers approximately 2.4 drum revolutions, allowing the input to contain multiple cutting-impact events as well as sustained motor-load variations. The 75-point interval corresponds to approximately 0.075 s, providing a dense temporal update while retaining sufficient contextual information. Each window sample comprised the Y-direction vibration acceleration and cutting-motor current signal, resulting in an input dimension of 2 × 1600. A total of 15,800 dual-channel window samples were generated from the 158 valid simulation records.

To prevent physically equivalent simulation records and their derived windows from being assigned to different data subsets, the records were partitioned using a stratified group-level strategy. Records with identical effective physical configurations were assigned to the same physical-condition group, and each group was associated with a unique identifier, physical_group_id. Before window generation, the 150 physical-condition groups were divided by class into 104 training groups, 23 validation groups, and 23 test groups, containing 108, 25, and 25 simulation records, respectively. Windows were then generated independently within each subset, yielding 10,800 training samples, 2500 validation samples, and 2500 test samples. All records and derived windows belonging to the same physical-condition group were restricted to a single subset. The normalization parameters were calculated exclusively from the training set, and class balancing and data augmentation were applied only to the training set. The detailed partitioning results are listed in Table 6.

## 4. Coal–Rock Cutting State Recognition Using CORRECT-Net

### 4.1. CORRECT-Net Architecture

To address the insufficient fusion of vibration-impact and current-load features and the difficulty in distinguishing adjacent coal–rock cutting states, a vibration–current multimodal recognition model, termed CORRECT-Net, was developed. CORRECT-Net denotes the Cross-Modal Correlation and Reliability-Enhanced Convolutional Transformer Network. The model consists of a SincNet-based adaptive frequency-band front end, a local convolutional feature extraction module, a Transformer temporal encoder, a residual importance-guided GATv2 cross-modal reasoning module, and a classifier. These components jointly model frequency-band-constrained features, local transient responses, long-range temporal dependencies, and cross-modal relationships between vibration and current signals.

#### 4.1.1. SincNet-Based Adaptive Frequency-Band Front End

To extract state-relevant frequency-band features from the vibration and current signals, a SincNet-based adaptive frequency-band front end was introduced at the multimodal input stage. SincNet parameterizes the convolution kernels in the first layer as band-pass filters and determines their frequency bands by learning the lower and upper cutoff frequencies. Compared with unconstrained convolution kernels, the resulting filters have more explicitly defined passbands [16]. Independent SincNet branches were employed for the vibration and current signals to extract frequency-band features associated with mechanical impacts and variations in the cutting load.

For the *k*th SincNet filter, the time-domain impulse response is jointly determined by its lower and upper cutoff frequencies as

(5) $$g_{k}\left[n\right]=\frac{2f_{h,k}}{f_{s}}\sin c\left(\frac{2\pi f_{h,k}n}{f_{s}}\right)-\frac{2f_{l,k}}{f_{s}}\sin c\left(\frac{2\pi f_{l,k}n}{f_{s}}\right)$$

where sinc(*x*) = sin(*x*)/*x*; *f_l,k_* and *f_h,k_* are the lower and upper cutoff frequencies of the *k*th band-pass filter, respectively, Hz; *f_s_* is the signal sampling frequency, which was set to 1000 Hz in this study; and *n* is the discrete-time index of the filter.

The cutoff frequencies satisfy 0 < *f_l,k_* < *f_h,k_* < *f_s_*/2. The upper cutoff frequency was parameterized as the sum of the lower cutoff frequency and a positive bandwidth to ensure valid lower and upper frequency bounds. To reduce spectral leakage caused by truncating the finite-length filter, a Hamming window was applied to smooth the filter as

(6) $${\tilde{g}}_{k}\left[n\right]=g_{k}\left[n\right]\cdot \omega \left[n\right]$$

where ${\tilde{g}}_{k}[n]$ is the windowed filter and *ω*[*n*] is the Hamming window function.

The vibration and current signals were processed using independent SincNet branches as

(7) $$\begin{array}{cc} S_{m}=X_{m}*{\tilde{G}}_{m} & m\in \left\{v,c\right\} \end{array}$$

where *X_m_* is the input signal of modality m; *v* and *c* denote the vibration and current modalities, respectively; ${\tilde{G}}_{m}$ is the learnable band-pass filter bank for modality m; * denotes one-dimensional convolution; and *S_m_* is the frequency-band feature output by SincNet.

After the frequency-band-constrained features were obtained, *S_m_* was fed into a one-dimensional convolutional feature extraction module to further capture local impacts, abrupt amplitude variations, and short-term cutting-load fluctuations as

(8) $$F_{m}=\Phi_{\mathrm{loc}}\left(S_{m}\right)$$

where *F_m_* denotes the local temporal features obtained after frequency-band-constrained filtering and local convolutional encoding of modality m, and Φ_loc_(·) denotes the local feature extraction module composed of one-dimensional convolution, batch normalization, a LeakyReLU activation function, and a pooling layer.

Through these operations, the raw vibration and current signals were transformed into frequency-band-constrained local temporal representations, providing the fundamental features required for subsequent local–global temporal modeling and reliability-aware cross-modal reasoning.

#### 4.1.2. Local–Global Temporal Encoder

The SincNet-based front end extracts frequency-band-constrained features from the raw vibration and current signals. However, complex coal–rock cutting states are characterized not only by local impacts and abrupt amplitude variations but also by sustained load variations and dynamic evolution across time steps. Therefore, a local–global temporal encoder was developed to jointly represent short-term disturbance features and long-range contextual information. Transformer architectures have been widely applied to time-series classification because their attention mechanisms can capture long-range temporal dependencies within individual modalities [17].

For the local temporal feature *F_m_* extracted from modality *m* by the SincNet-CNN branch, global average pooling and linear projection were applied to obtain the local feature vector as

(9) $$I_{m}=\mathrm{LN}\left(W_{l}\cdot \mathrm{GAP}\left(F_{m}\right)\right)$$

where *I_m_* denotes the local feature vector of modality *m*; GAP(·) denotes the global average pooling operation; *W_l_* is the linear projection matrix; and LN(·) denotes the layer normalization operation.

To capture the evolution of the cutting state over a longer temporal range, *F_m_* was fed into a temporal Transformer encoder. The global temporal encoding process was expressed as

(10) $$H_{m}=\mathrm{Transformer}\left(F_{m}+P\right)$$

where *H_m_* = [*h_m_*_,1_,*h_m_*_,2_,…,*h_m_*_,*T*′_] denotes the encoded temporal contextual feature sequence; *h_m_*_,*t*_ is the contextual feature of modality *m* at the *t*-th time step; *T*′ is the number of time steps after downsampling; and *P* denotes the positional encoding.

To emphasize the key time segments associated with the cutting state, attention pooling was applied to the global temporal features. The attention weight at the *t*-th time step and the resulting global temporal feature vector were calculated as

(11) $$\beta_{m,t}=\frac{\exp\left(w_{a}^{T}\tanh\left(W_{a}h_{m,t}\right)\right)}{\sum_{q=1}^{T^{\prime }}\exp\left(w_{a}^{T}\tanh\left(W_{a}h_{m,q}\right)\right)}\;gm=\sum_{t=1}^{T^{\prime }}\beta_{m,t}h_{m,t}$$

where *β_m_*_,*t*_ denotes the attention weight assigned to the *t*-th time step of modality m; *W_a_* is the learnable feature transformation matrix; *w_a_* is the learnable attention vector; *q* is the time-step index used for attention-weight normalization; and *g_m_* denotes the global temporal feature vector of modality *m*.

The local–global temporal encoder extracted local impact features and global evolution features from the vibration and current modalities, allowing the model to characterize both short-term transient responses and long-range load variations. The resulting vibration local feature, vibration global feature, current local feature, and current global feature were not directly concatenated and fed into the classifier. Instead, these four feature vectors were constructed as four nodes of the subsequent cross-modal graph reasoning module to model the dynamic interactions among different features.

#### 4.1.3. Residual Importance-Guided GATv2 Cross-Modal Reasoning

During complex coal–rock cutting, the vibration and current signals exhibit different sensitivities to different cutting states. The vibration signal mainly reflects gangue impacts, coal–rock fragmentation, and local mechanical disturbances, whereas the current signal primarily characterizes variations in the cutting load and motor torque demand. Direct feature concatenation cannot explicitly represent the interactions among different features or dynamically adjust their contributions to the fusion result according to each input sample. Previous studies have shown that GATv2 can dynamically calculate graph attention coefficients from node features and is suitable for modeling relationships among multisource features [18]. Therefore, a residual importance-guided GATv2 cross-modal reasoning module was developed. The vibration local feature, vibration global feature, current local feature, and current global feature were constructed as graph nodes. Adaptive node-importance weighting, dynamic graph attention propagation, and residual interaction fusion were then applied to jointly model the multimodal features.

The local and global features of the vibration and current modalities were mapped to a common feature dimension and constructed as a fully connected four-node graph as

(12) $$N=\left[I_{v},g_{v},I_{c},g_{c}\right]\;G=\left(V,E\right),V=\left\{v_{l},v_{g},c_{l},c_{g}\right\}$$

where *N* is the graph-node feature set; *I_v_* and *g_v_* denote the local and global features of the vibration modality, respectively; *I_c_* and *g_c_* denote the local and global features of the current modality, respectively; *G* is the constructed feature graph; *V* is the node set; *v_l_* and *v_g_* are the local and global vibration nodes, respectively; *c_l_* and *c_g_* are the local and global current nodes, respectively; and *E* is the edge set connecting the nodes.

To adaptively adjust the contribution of each node to the current input sample, a node reliability weight was introduced as

(13) $$r_{i}=\frac{\exp\left(f_{r}\left(n_{i}\right)\right)}{\sum_{j=1}^{4}\exp\left(f_{r}\left(n_{j}\right)\right)}$$

where *r_i_* is the reliability weight of the *i*-th node; *n_i_* is the feature of the *i*-th node; j is the node index used for weight normalization; and *f_r_*(·) is the reliability estimation function.

GATv2 was then used to calculate the dynamic attention score between nodes as

(14) $$e_{ij}^{h}=a_{h}^{T}\mathrm{LeakyReLU}\left(W_{l}^{h}n_{i}+W_{r}^{h}n_{j}\right)$$

where *e_ij_^h^* is the attention score from node *j* to node *i* under the *h*-th attention head; *h* is the attention-head index; *a_h_* is the learnable attention vector of the *h*-th attention head; *W_l_^h^* and *W_r_^h^* are learnable feature-transformation matrices for the target and source nodes, respectively; and superscript *T* denotes matrix transposition.

The node reliability weights were further incorporated into the attention normalization as

(15) $$\alpha_{ij}^{h}=\frac{\exp\left(e_{ij}^{h}+\log\left(r_{j}+\varepsilon \right)\right)}{\sum_{q\in N_{i}}\exp\left(e_{iq}^{h}+\log\left(r_{q}+\varepsilon \right)\right)}$$

where *α_ij_^h^* is the contribution weight from node *j* to node *i* under the *h*-th attention head; *N_i_* is the neighborhood set of node *i*; *q* is the index of a neighboring node used for attention normalization; *r_j_* and *r_q_* are the reliability weights of nodes *j* and *q*, respectively; and *ε* is a small positive constant introduced to avoid an undefined logarithm when a node weight is zero.

Through this design, the attention allocation simultaneously considered the relationships between nodes and their relative contributions. The neighborhood information of each node was aggregated using multiple attention heads, and the outputs of all attention heads were concatenated to obtain the node representation. The resulting node representations were then mapped to obtain the cross-modal graph representation as

(16) $$\begin{array}{l} {\tilde{n}}_{i}^{h}=\sum_{j\in N_{i}}\alpha_{ij}^{h}W_{r}^{h}n_{j}\\ {\tilde{n}}_{i}={\|}_{h=1}^{H}{\tilde{n}}_{i}^{h} \end{array}\;z_{g}=\sum_{i=1}^{4}r_{i}W_{o}{\tilde{n}}_{i}$$

where ${\tilde{n}}_{i}^{h}$ is the node feature produced by the *h*-th attention head; *H* is the number of attention heads; || denotes feature concatenation; ${\tilde{n}}_{i}$ is the node representation obtained by concatenating the outputs of all attention heads; *W_o_* is the graph-readout projection matrix; *i* is the node index; and *z_g_* is the cross-modal graph representation.

To prevent graph propagation from weakening the effective discriminative information contained in the original modalities, a residual interaction fusion vector was further constructed as

(17) $$z=\left[g_{v}\left\|g_{c}\right.\left\|z_{g}\right.\left\|\left|g_{v}-g_{c}\right|\right.\left\|g_{v}⊙g_{c}\right.\right]$$

where *g_v_* and *g_c_* are the global temporal feature vectors of the vibration and current modalities, respectively; |*g_v_* − *g_c_*| is the absolute difference between the two modality features; *g_v_* ⊙ *g_c_* is the element-wise interaction between the two modality features; and *z* is the final residual interaction fusion vector.

This fusion strategy retained the original modality features while incorporating the cross-modal relational information obtained through graph reasoning. Finally, the fused feature was fed into the classifier, and the predicted probabilities of the four coal–rock cutting states were obtained through the Softmax function as

(18) $$\hat{y}=\mathrm{Soft}\max\left(f_{\theta }\left(z\right)\right)$$

where *ŷ* is the predicted probability vector for the four coal–rock cutting states; *f_θ_*(·) is the classification mapping function; and *θ* denotes the learnable parameters of the classifier.

Overall, the module jointly modeled the vibration and current features through node-weight adjustment, graph attention propagation, and residual interaction fusion. Unlike a simple serial combination or direct concatenation of SincNet, Transformer, and GATv2 features, CORRECT-Net explicitly constructs four semantic nodes from the vibration-local, vibration-global, current-local, and current-global representations. GATv2 is then used to model the dynamic interactions among these four nodes, while an importance-estimation branch generates sample-dependent reliability weights for adaptive node reweighting. A residual connection further preserves the original multimodal information during graph reasoning. Consequently, the proposed fusion strategy performs reliability-aware interaction modeling across modalities and temporal scales rather than uniform feature concatenation. The main architectural parameters of CORRECT-Net are summarized in Table 7.

#### 4.1.4. Interpretability Analysis of Multimodal Representations

**Figure 7:**
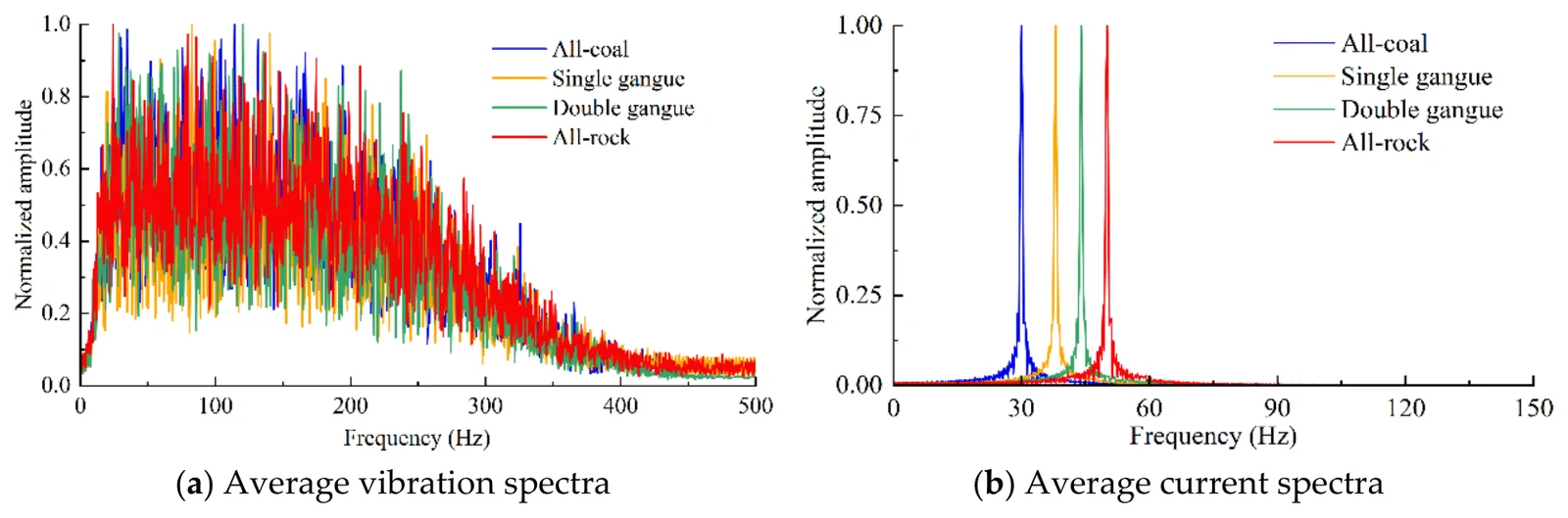
Average normalized spectra of vibration and current signals under different cutting states.

To analyze the multimodal discriminative features learned by CORRECT-Net, visualization was performed from three perspectives: frequency-domain responses, temporal occlusion sensitivity, and deep-feature distributions. All results were aggregated by class using the test-set samples to reduce the influence of variations among individual samples. The class-averaged normalized spectra of the vibration and current signals under different coal–rock cutting states are shown in Figure 7.

As shown in Figure 7a, the vibration signals of all four classes exhibit broadband distributions over 0–500 Hz. However, differences are observed in the normalized spectral profiles within the intermediate-frequency range and the high-frequency attenuation region. In Figure 7b, the current spectra are primarily concentrated within 30–55 Hz, and the main peak positions and adjacent frequency components vary among the different cutting states. Overall, the two modalities exhibit distinct frequency-domain characteristics, providing complementary information for subsequent multimodal fusion.

**Figure 8:**
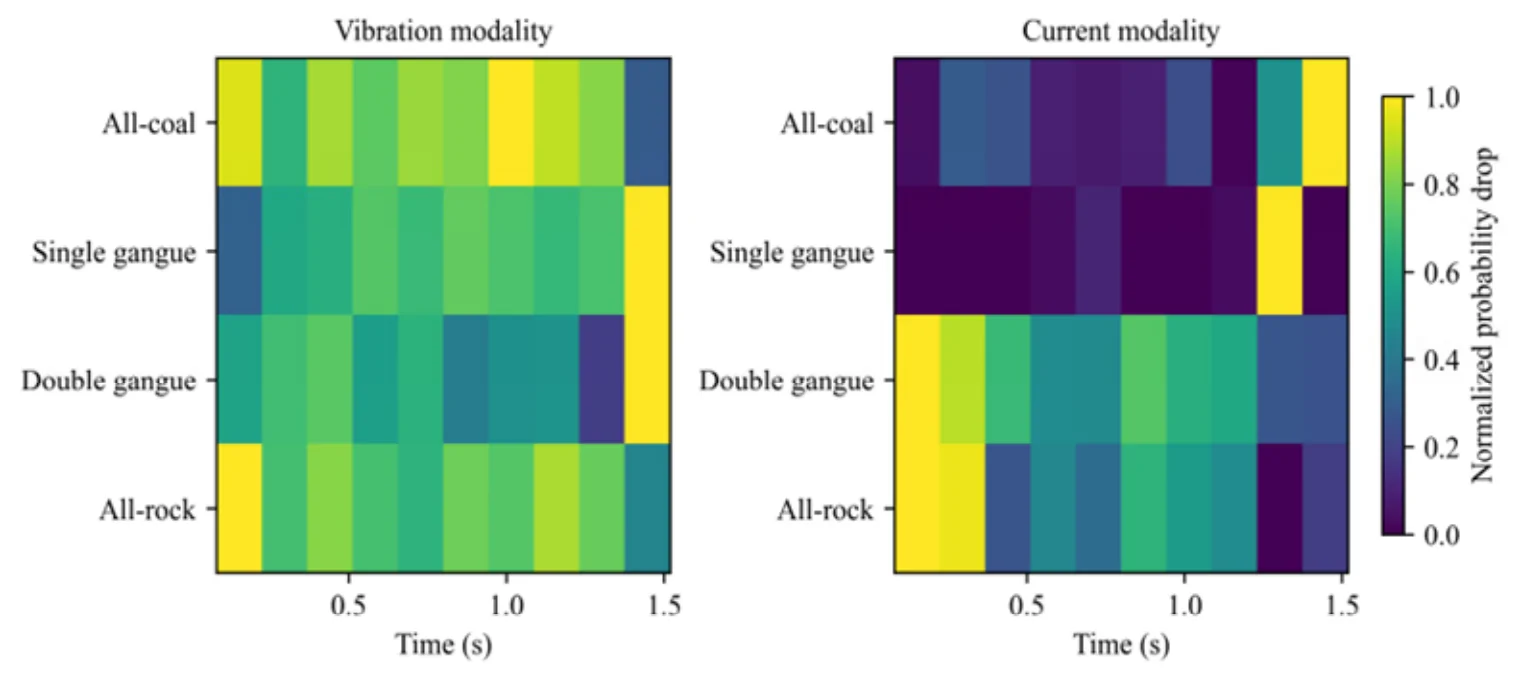
Temporal occlusion sensitivity of vibration and current modalities under different cutting states.

Temporal occlusion analysis was conducted to evaluate the contributions of different time segments to the classification results. Local segments of the input signals were sequentially masked, and the reduction in the predicted probability of the true class was used as the sensitivity measure. The results are shown in Figure 8.

As shown in Figure 8, the temporal sensitivity distributions of the vibration and current modalities differ across the cutting states. The vibration modality exhibits relatively high sensitivity over multiple time segments, whereas the highly sensitive regions of the current modality are more concentrated. These results indicate that the two signals provide different discriminative information in the temporal dimension and therefore contribute complementary features to multimodal fusion.

**Figure 9:**
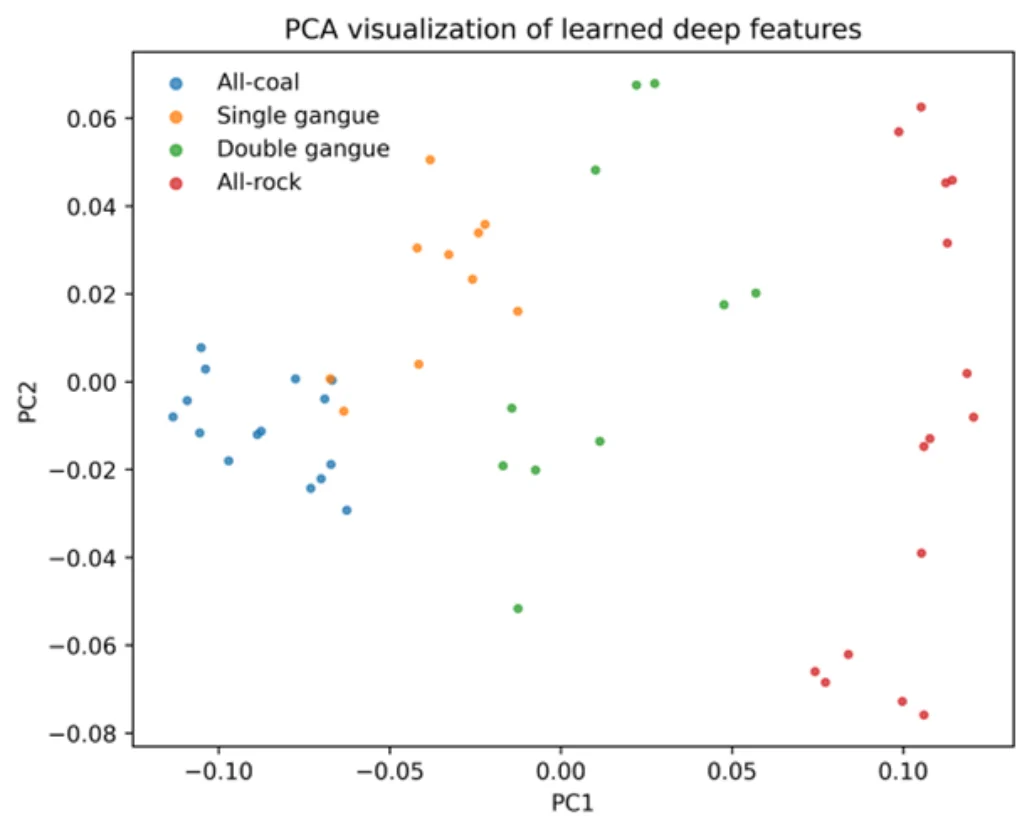
PCA visualization of deep multimodal features learned by CORRECT-Net.

To examine the distribution of the deep fused features, principal component analysis (PCA) was applied to project the test-set features into a two-dimensional space, as shown in Figure 9.

The All-coal and All-rock samples form relatively compact distributions, whereas partial overlap remains between the Single gangue layer and Double gangue layer samples. Overall, the four classes exhibit a certain degree of class-wise clustering in the two-dimensional projection, although confusion remains between the two adjacent gangue-bearing states.

Figure 7, Figure 8 and Figure 9 collectively show that the vibration and current modalities exhibit different characteristics in terms of frequency-domain distributions and temporal sensitivity, thereby providing complementary information for cutting-state recognition. The fused deep features show discernible class-wise clustering in the two-dimensional projection, although local overlap remains between the Single gangue layer and Double gangue layers classes. Overall, CORRECT-Net jointly represents mechanical vibration responses and motor-load variations, providing discriminative feature representations for complex coal–rock cutting state recognition.

### 4.2. Analysis of Recognition Results

CORRECT-Net was trained using PyTorch 2.0.0 with the AdamW optimizer. The initial learning rate was 8 × 10^−5^, the batch size was 16, the weight-decay coefficient was 3 × 10^−4^, the dropout rate was 0.20, the label-smoothing coefficient was 0.01, and the maximum number of training epochs was 180. The validation set was used for model selection, whereas the test set was used only for the final evaluation. Each simulation experiment was independently repeated five times, and the results are reported as the mean ± standard deviation.

To evaluate the sensitivity of CORRECT-Net to the window parameters, a controlled experiment was conducted in which the physical-condition-group partition, model architecture, training procedure, and evaluation metrics were kept unchanged, while only the window length or window-start interval was varied. Five settings were evaluated, and the results are summarized in Table 8.

As shown in Table 8, the 1600-point window with an average 75-point window-start interval achieved the highest observed recognition performance, with an accuracy of 96.20%, a Macro-F1 score of 95.14%, and a hazardous-condition miss rate of 0.58%. The relatively small differences between the 1600- and 2000-point windows indicate that the model remained stable within this range. Overall, the selected 1600/75 setting provides a favorable balance among recognition performance, frequency resolution, temporal context, and update density.

Accuracy, macro-precision, macro-recall, macro-F1 score, and the confusion matrix were used to evaluate the recognition performance. The macro-averaged metrics were calculated as the arithmetic means of the corresponding metrics for the four classes. The class-specific miss rate and false-alarm rate reported in Table 9 were defined as *FN_i_*/(*TP_i_* + *FN_i_*) and *FP_i_*/(*FP_i_* + *TN_i_*), respectively. In addition, the proportion of Single gangue layer, Double gangue layers, and All-rock windows misclassified as All-coal was defined as the hazardous-condition miss rate. The hazardous-condition miss rate was calculated as

(19) $$R_{\mathrm{miss}}=\frac{N_{SG\to AC}+N_{DG\to AC}+N_{AR\to AC}}{N_{SG}+N_{DG}+N_{AR}}\times 100\%$$

where *R_miss_* denotes the hazardous-condition miss rate; *N_SG→AC_*, *N_DG→AC_*, and *N_AR→AC_* denote the numbers of Single gangue layer, Double gangue layers, and All-rock samples misclassified as All-coal, respectively; and *N_SG_*, *N_DG_*, and *N_AR_* denote the corresponding numbers of hazardous-condition samples in the test set. All metrics reported in this section were calculated using the test-set samples.

#### 4.2.1. Analysis of Overall Recognition Performance

CORRECT-Net was independently trained and evaluated five times on the 2500 test windows and achieved an accuracy of 96.20% ± 0.11%. The class-specific performance metrics obtained from the five independent runs are presented in Table 9.

As presented in Table 9, CORRECT-Net achieved a macro-precision of 93.21% ± 0.38%, a macro-recall of 97.31% ± 0.04%, and a macro-F1 score of 95.14% ± 0.21%. The recall values for All-coal and All-rock were both 100.00%, indicating that all true samples from these two classes were correctly recognized across the five independent runs. The precision of All-coal was 87.77% ± 2.44%, indicating that a small number of gangue-bearing samples were still misclassified as All-coal.

The recall and F1-score for Single gangue layer were 93.00% ± 0.00% and 92.08% ± 0.27%, respectively, which were lower than the corresponding values of 96.25% ± 0.17% and 97.44% ± 0.18% for Double gangue layers. This result indicates that the classification errors were mainly concentrated between the two adjacent gangue-bearing states. Overall, CORRECT-Net achieved an accuracy of 96.20% ± 0.11% and a hazardous-condition miss rate of 0.58% ± 0.13% on the current test set. The model therefore exhibited relatively high and stable recognition performance, although the discrimination between adjacent gangue-bearing states could be further improved.

**Figure 10:**
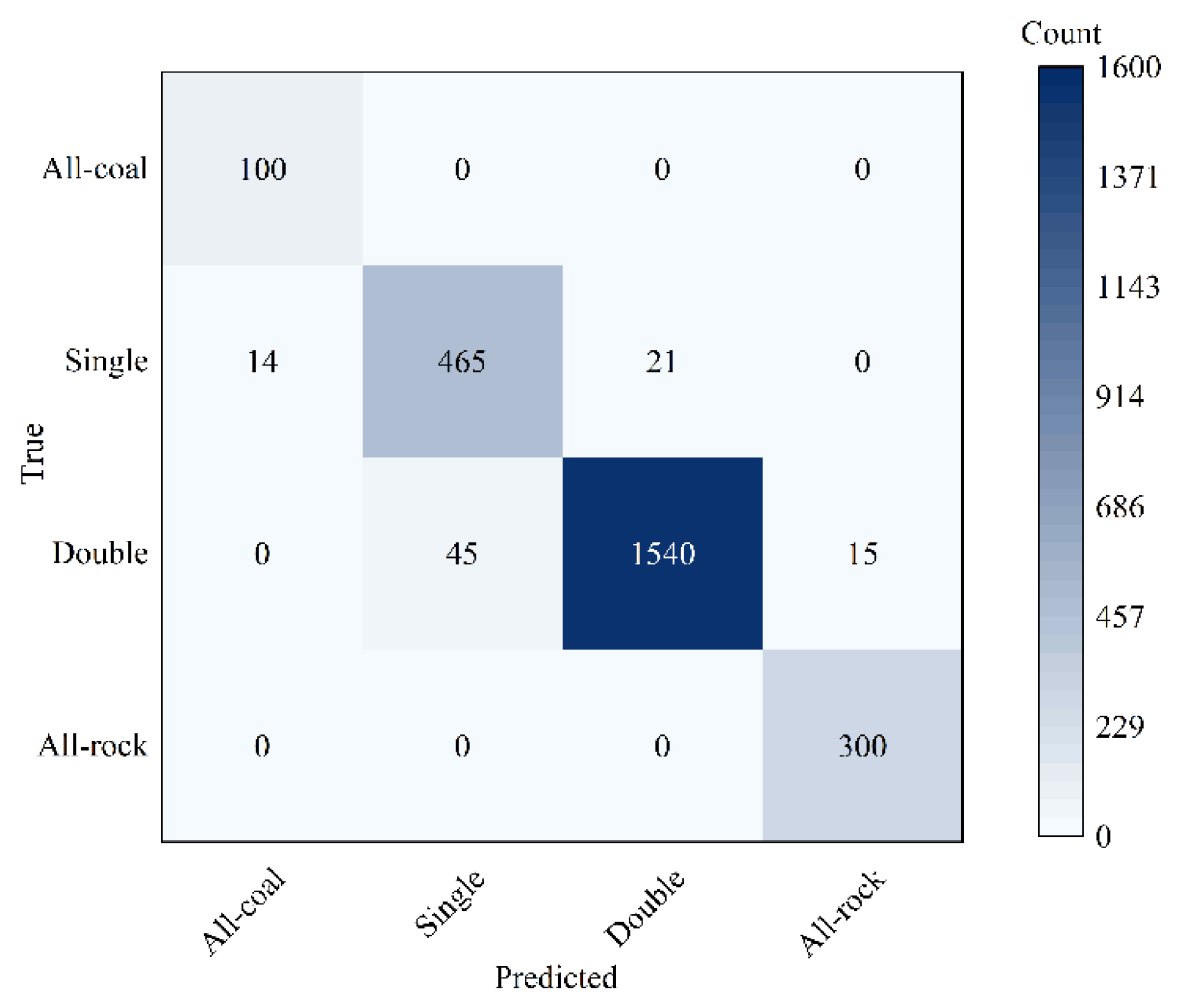
Mean confusion matrix of CORRECT-Net on the test set over five independent runs.

To further examine the misclassification relationships among the four classes, the mean confusion matrix over the five independent runs was plotted, as shown in Figure 10.

Figure 10 shows that all All-coal and All-rock samples were correctly recognized. For the Single gangue layer class, an average of 465 samples were correctly classified, whereas 14 samples were misclassified as All-coal and 21 were misclassified as Double gangue layers. For the Double gangue layers class, an average of 1540 samples were correctly classified, whereas 45 samples were misclassified as Single gangue layer and 15 were misclassified as All-rock.

The misclassifications were mainly concentrated between Single gangue layer and Double gangue layers, indicating that the features of these two adjacent gangue-bearing states retained a certain degree of similarity. Among the 2400 hazardous-condition test samples, only an average of 14 Single gangue layer samples were misclassified as All-coal, corresponding to a hazardous-condition miss rate of 0.58% ± 0.13%. Overall, CORRECT-Net maintained high recognition accuracy while keeping the proportion of hazardous conditions misclassified as All-coal at a relatively low level.

#### 4.2.2. Analysis of Ablation Results

To analyze the effects of the individual modules under the present progressive configuration, four model variants were evaluated: M-Base, M-Sinc, M-Trans, and the complete CORRECT-Net. All models used the same dataset partition and training strategy and were independently run five times. The results are presented in Table 10.

As shown in Table 10, the recognition performance progressively improved as the modules were sequentially incorporated under the present configuration. Compared with M-Base, the introduction of SincNet increased the Accuracy and Macro-F1 by 3.88 and 4.46 percentage points, respectively, while reducing the hazardous-condition miss rate from 1.46% ± 0.13% to 1.04% ± 0.08%. After the Transformer temporal encoder was further incorporated, the Accuracy and Macro-F1 increased to 93.24% ± 0.17% and 91.57% ± 0.25%, respectively. With ResGATv2 fusion, the complete CORRECT-Net achieved an Accuracy of 96.20% ± 0.11% and a Macro-F1 of 95.14% ± 0.21%, while the hazardous-condition miss rate was further reduced to 0.58% ± 0.13%.

Overall, compared with M-Base, the complete CORRECT-Net increased the Accuracy and Macro-F1 by 8.80 and 10.57 percentage points, respectively, and reduced the hazardous-condition miss rate by 0.88 percentage points. These results indicate that, under the present progressive configuration, the sequential combination of frequency-band-constrained feature extraction, temporal encoding, and graph-based fusion continuously improved the recognition performance and the control of hazardous-condition misses.

#### 4.2.3. Analysis of Comparative Recognition Results

To compare the recognition performance of different models and input modalities, SVM, 1D-CNN, Bi-LSTM, and TCN were selected as baseline models. All models used the same dataset partition, preprocessing procedure, and evaluation metrics and were independently run five times. The results are presented in Table 11.

As presented in Table 11, the vibration modality achieved higher accuracy and macro-F1 scores than the corresponding current modality for all deep-learning models. This result indicates that, under the present experimental conditions, the vibration signal provided more discriminative information for coal–rock cutting state recognition. After the current modality was incorporated, the accuracy and macro-F1 scores of all models increased, whereas their hazardous-condition miss rates decreased. These results demonstrate that the vibration and current signals provided complementary information for distinguishing different cutting states.

Under the vibration–current multimodal setting, CORRECT-Net achieved an accuracy of 96.20% ± 0.11% and a macro-F1 score of 95.14% ± 0.21%. Compared with the multimodal 1D-CNN, TCN, and Bi-LSTM models, CORRECT-Net improved the accuracy by 8.80, 4.00, and 2.08 percentage points, respectively, and improved the macro-F1 score by 10.57, 6.53, and 3.61 percentage points, respectively. Its hazardous-condition miss rate was reduced to 0.58% ± 0.13%, which was lower than those of the other comparison models.

Overall, CORRECT-Net achieved the highest accuracy and macro-F1 score among the compared models under the vibration-only, current-only, and vibration–current multimodal settings. The best overall performance was obtained using the vibration–current input, indicating that frequency-band-constrained feature extraction, temporal modeling, and cross-modal graph fusion improved the recognition of complex coal–rock cutting states under the present conditions.

To further assess the computational efficiency and potential for online deployment, the parameter count, FP32 weight storage, FLOPs, and batch-1 inference latency of CORRECT-Net and the deep-learning baseline models were evaluated under the same input configuration, as summarized in Table 12.

CORRECT-Net contains 1,031,111 trainable parameters, corresponding to approximately 3.93 MiB of FP32 weight storage, and requires approximately 0.195 GFLOPs for one forward inference. Its mean batch-1 inference latency was 7.91 ms in our CPU benchmark. Although CORRECT-Net is more complex than the lightweight 1D-CNN and TCN in terms of parameter count, its measured latency is substantially shorter than the approximately 75 ms window-update interval used in the present recognition procedure. Therefore, the results support the preliminary feasibility of online inference under the present input configuration. However, because the current latency benchmark was conducted on a general-purpose CPU rather than a specific underground embedded controller, verified embedded deployment cannot yet be claimed, and device-level validation on representative underground hardware remains necessary.

#### 4.2.4. Robustness Analysis Under Signal Perturbations and Modality Absence

To evaluate the recognition performance of CORRECT-Net under signal perturbations and modality-absence conditions, Gaussian noise, amplitude drift, random modality dropout, and single-modality absence were separately introduced into the test set. The standard deviation of Gaussian noise was set to 0.01, 0.03, and 0.05. Amplitude drift was simulated by randomly scaling the signal amplitude within ±10%. For random modality dropout, either the vibration or current modality was set to zero for each window with a probability of 0.10. For single-modality absence, the corresponding modality was consistently set to zero while all model parameters remained unchanged. The results are presented in Table 13.

As presented in Table 13, when the standard deviation of the Gaussian noise increased from 0.01 to 0.05, the Accuracy decreased from 95.68% ± 0.15% to 92.76% ± 0.24%, while the Macro-F1 decreased from 94.68% ± 0.43% to 90.59% ± 0.13%. At a noise standard deviation of 0.05, the Accuracy was 3.44 percentage points lower than that under the clean condition but remained above 92%. Under random modality dropout and amplitude drift, the Accuracy values were 93.00% ± 0.29% and 93.48% ± 0.27%, respectively, indicating that the model retained relatively stable recognition performance under the tested perturbation settings.

Under modality absence, the Accuracy and Macro-F1 decreased to 86.56% ± 0.37% and 83.08% ± 0.34%, respectively, when the vibration modality was absent, while the hazardous-condition miss rate increased to 3.46% ± 0.24%. When the current modality was absent, the Accuracy and Macro-F1 were 92.44% ± 0.25% and 90.94% ± 0.46%, respectively. The greater performance degradation caused by vibration-modality absence indicates that the model was more sensitive to the loss of vibration information. Under the present recognition conditions, the vibration signal therefore provided the primary discriminative information, whereas the current signal supplied complementary cutting-load information.

Overall, CORRECT-Net retained stable recognition performance under the tested Gaussian noise, amplitude drift, and random modality-dropout conditions. Although the model exhibited a certain degree of tolerance to single-modality absence, its performance was highest when both modalities were available, further indicating that the fusion of vibration and current information improved the stability of complex coal–rock cutting state recognition.

## 5. Experimental Validation and Analysis of Transfer Results

**Figure 11:**
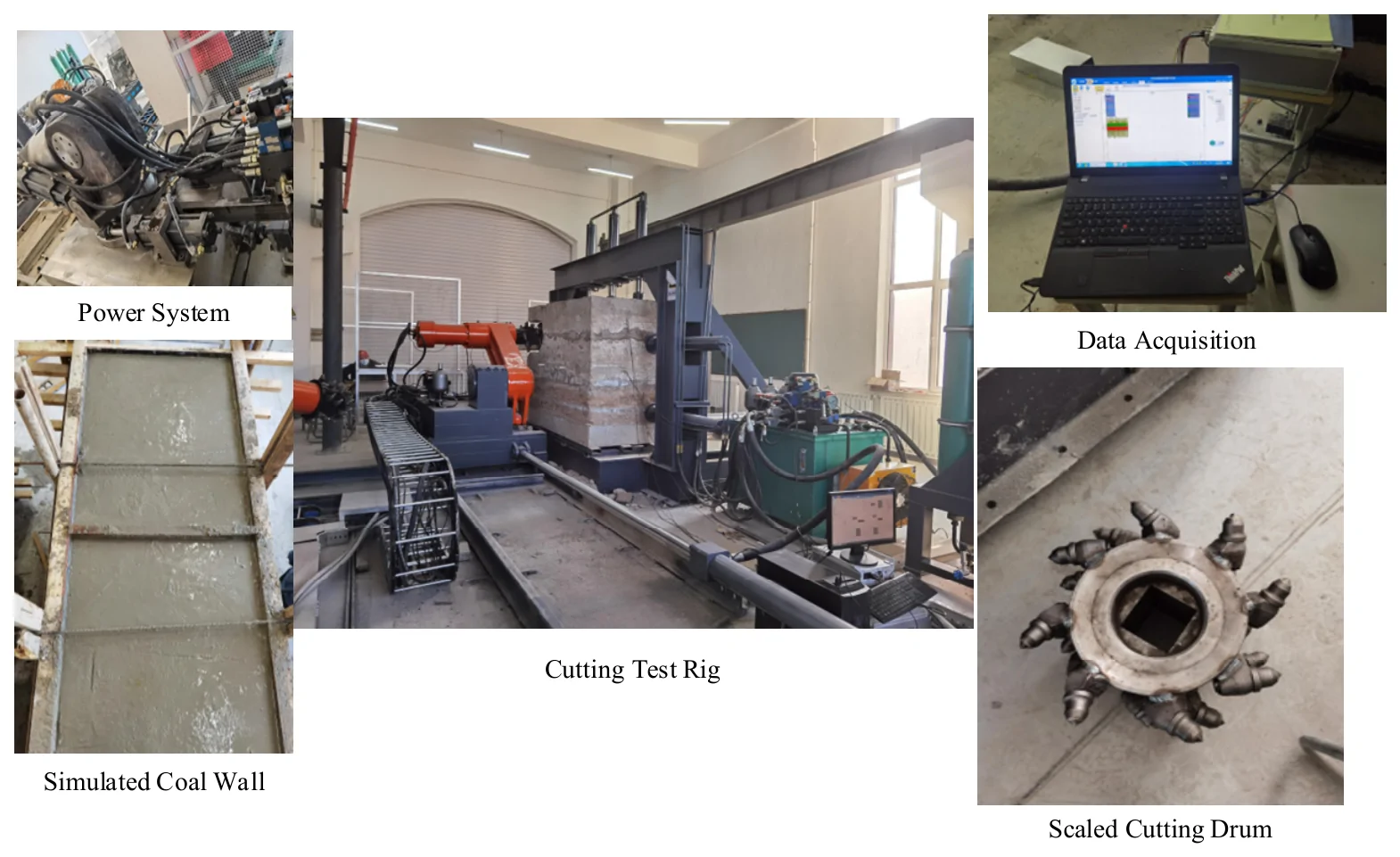
Similarity-based physical test bench for shearer cutting.

To evaluate the simulation-to-experiment transfer recognition performance of CORRECT-Net under experimental cutting conditions, a similarity-based shearer cutting test bench was constructed based on similarity theory, as shown in Figure 11. The test bench consisted of a similar-material coal wall, a power system, a cutting system, and a data acquisition system. A wireless accelerometer was installed at the connection between the helical cutting drum and the ranging arm to acquire vibration signals, while a current sensor was installed at the output terminal of the variable-frequency drive to acquire the cutting-motor current. The vibration and current signals were synchronously acquired using a common time reference, and both channels were sampled at 1000 Hz to maintain their temporal correspondence.

The cutting depth was set to 315 mm, the haulage speed was set to 4 m/min, and the cutting-drum rotational speed was set to 90 r/min. Four experimental cutting states, namely All-coal, Single gangue layer, Double gangue layers, and All-rock, were constructed by adjusting the firmness coefficients and volume ratios of the coal and gangue materials in the similar-material coal wall. For the All-coal condition, the coal firmness coefficient was *f* = 1.4, and the coal–rock volume ratio was 1:0. For the Single gangue layer condition, the coal and gangue firmness coefficients were *f* = 1.4 and *f* = 5.1, respectively, and the coal–rock volume ratio was 3:1. For the Double gangue layers condition, the coal firmness coefficient was *f* = 1.4, the firmness coefficients of the two gangue layers were *f* = 3.5 and *f* = 5.1, respectively, and the coal–rock volume ratio was 2:2:1. For the All-rock condition, the rock firmness coefficient was *f* = 7.4, and the coal–rock volume ratio was 0:1. Five independent cutting tests were conducted for each cutting state. Each test was independently initiated, and signals were collected from a different cutting segment. A total of 20 experimental records were obtained, and each independent experimental run was assigned a unique identifier, run_id.

The 20 experimental records were processed using the same preprocessing and window-construction procedures as those applied to the simulation data. The vibration and cutting-motor current signals were used as the dual-channel input. After the startup transient was removed, 300 window samples were generated, comprising 75 samples for each cutting state.

To quantify the domain discrepancy, the simulation and experimental signals after removal of the initial 1 s startup segment were compared using amplitude, spectral, and transient indicators. For the vibration signals, the RMS discrepancy between the simulation and experimental domains ranged from 3.37% to 5.74% across the four cutting states. For the current signals, the corresponding RMS discrepancy ranged from 4.25% to 5.52%. In the frequency domain, the spectral centroid of the experimental vibration signals was 10.30–13.19% lower than that of the simulated signals, with an average reduction of approximately 11.38%. For the current signals, the dominant frequency remained at 49.80 Hz in both domains, whereas the energy proportion within 30–55 Hz decreased from 99.71–99.86% in simulation to 97.03–97.96% in the experimental signals, indicating a modest increase in broadband and harmonic components.

The transient indicators also showed domain-dependent differences. On average, the vibration kurtosis and crest factor decreased by approximately 5.99% and 12.42%, respectively, whereas the current kurtosis and crest factor increased by approximately 7.07% and 19.80%. These discrepancies are consistent with differences in structural transmission paths, damping, boundary conditions, sensor mounting, random coal–rock fragmentation, motor-drive harmonics, and measurement noise between the simulation and physical test systems. During fine-tuning, the SincNet frequency-band parameters, local convolutional representations, Transformer temporal features, reliability-estimation branch, GATv2 cross-modal interactions, and classifier decision boundaries are jointly updated. Consequently, the adaptation procedure compensates not only for amplitude-scale differences but also for spectral redistribution, transient-shape variations, and changes in the relative reliability of the vibration and current modalities.

To prevent windows derived from the same experimental run from being included in both the adaptation and test sets, an independent-run-based five-fold transfer evaluation was performed using run_id. In each fold, one complete run from each cutting state was selected as the test set, resulting in 60 test windows. The remaining 16 runs formed an adaptation pool containing 240 candidate windows. The experimental-data adaptation ratios were set to 0%, 5%, 10%, 20%, and 50%, corresponding to 0, 12, 24, 48, and 120 adaptation windows per fold, respectively. These settings provided 0, 3, 6, 12, and 30 adaptation windows for each class. The adaptation windows were sampled in a class-balanced manner. Within each fold, the test runs remained unchanged across the different adaptation ratios and were excluded from model fine-tuning and parameter estimation. For each fold, the simulation-trained CORRECT-Net was used for parameter initialization. Fine-tuning was performed for 40 epochs using AdamW with a learning rate of 2 × 10^−5^, a batch size of 16, a weight decay of 3 × 10^−4^, and label smoothing of 0.01. All trainable modules, including the SincNet front end, local convolutional layers, Transformer encoder, reliability-estimation branch, GATv2 fusion module, and classifier, were jointly updated, and no layer was frozen. The independent experimental runs assigned to the test set were excluded from all fine-tuning and parameter-estimation procedures. The transfer recognition results are presented in Table 14.

As presented in Table 14, without experimental-data adaptation, the five-fold mean Accuracy and Macro-F1 were 91.33% ± 5.19% and 91.32% ± 5.26%, respectively. These results indicate that the model pretrained on the simulation data correctly recognized most experimental windows, although its performance varied among the different test runs. The transfer recognition performance generally improved as the proportion of experimental samples used for adaptation increased. At adaptation ratios of 5% and 10%, the Accuracy increased to 93.67% ± 3.98% and 95.00% ± 3.12%, respectively, while the Macro-F1 increased to 93.60% ± 4.09% and 94.98% ± 3.13%, respectively.

When the adaptation ratio reached 20%, the Accuracy and Macro-F1 were 96.33% ± 0.75% and 96.32% ± 0.75%, respectively. Compared with the condition without experimental-data adaptation, both metrics increased by 5.00 percentage points, while the inter-fold variability was markedly reduced. When the adaptation ratio was further increased to 50%, the Accuracy and Macro-F1 reached 98.67% ± 1.39% and 98.68% ± 1.38%, respectively. The hazardous-condition miss rate decreased from 3.11% ± 3.72% without adaptation to 0.00% ± 0.00% at the 20% and 50% adaptation ratios. Thus, under these two adaptation settings, no hazardous-condition windows were observed to be misclassified as All-coal in the current five-fold test results.

Overall, adaptation using a limited number of experimental samples improved the recognition of experimental-domain signals and reduced the risk of hazardous-condition misses. These results indicate that CORRECT-Net achieved measurable experimental-domain adaptability within the present test bench, sensor configuration, and four experimental cutting states.

## 6. Conclusions

(1) The original simulation records were examined for physical equivalence, and physical-condition group-level partitioning was adopted to construct the training, validation, and test sets. On the 2500 test windows, CORRECT-Net achieved an accuracy of 96.20% ± 0.11%, a macro-F1 score of 95.14% ± 0.21%, and a hazardous-condition miss rate of 0.58% ± 0.13%. Compared with the vibration–current multimodal 1D-CNN, TCN, and Bi-LSTM models, the accuracy was improved by 8.80, 4.00, and 2.08 percentage points, respectively. These results indicate that CORRECT-Net effectively integrated vibration-impact and current-load features and improved the recognition of complex coal–rock cutting states.

(2) A progressive ablation study and input-perturbation tests were conducted to evaluate the contributions of the individual modules and the robustness of the model. With the sequential incorporation of SincNet, Transformer, and residual GATv2, the accuracy increased from 87.40% ± 0.26% to 96.20% ± 0.11%, the macro-F1 score increased from 84.57% ± 0.34% to 95.14% ± 0.21%, and the hazardous-condition miss rate decreased from 1.46% ± 0.13% to 0.58% ± 0.13%. When the standard deviation of the Gaussian noise was 0.05, the model retained an accuracy of 92.76% ± 0.24%. When the vibration and current modalities were absent, the accuracy values were 86.56% ± 0.37% and 92.44% ± 0.25%, respectively. These results indicate that the progressive combination of the three modules improved the recognition performance and that the model retained relatively stable performance under the tested noise and modality-absence conditions. The vibration modality provided the primary discriminative information, whereas the current modality supplied complementary cutting-load information.

(3) An independent-run-based five-fold transfer evaluation was conducted using five independent experimental runs for each cutting state. Without experimental-data adaptation, the accuracy and macro-F1 score were 91.33% ± 5.19% and 91.32% ± 5.26%, respectively. After adaptation using 20% of the experimental windows, these values increased to 96.33% ± 0.75% and 96.32% ± 0.75%, respectively. With a 50% adaptation ratio, they further increased to 98.67% ± 1.39% and 98.68% ± 1.38%, respectively. These results indicate that adaptation using a limited number of experimental samples improved recognition performance in the experimental domain and reduced performance variations among different experimental runs under the present simulation and experimental conditions. The applicability of these findings is limited to the present simulation settings, test-bench configuration, sensor arrangement, and four cutting states; further validation under different geological conditions and field operating environments is required before broader generalization.

## Figures and Tables

**Table 1:** Contact parameters of the gear pairs in the shearer cutting unit.

Gear Pair	Contact Stiffness /(N·mm^−1^)	Damping Coefficient/(N·s·mm^−1^)	Max Penetration/mm	Nonlinear Exponent
Gear 1 & Gear 2	788,530	788.53	0.1	1.5
Gear 2 & Gear 3	890,370	890.37	0.1	1.5
Gear 3 & Gear 4	788,530	788.53	0.1	1.5
Gear 4 & Gear 5	788,530	788.53	0.1	1.5
Gear 5 & Gear 6	964,460	964.46	0.1	1.5
Gear 7 & Gear 8	891,780	891.78	0.1	1.5
Gear 8 & Gear 9	1,019,000	1019.00	0.1	1.5

**Table 2:** Physical and mechanical properties of coal and gangue.

Material Type	Elastic Modulus (GPa)	Density (kg/m^3^)	Tensile Strength (MPa)	Compressive Strength (MPa)	Poisson’s Ratio	Protodyakonov Coefficient (*f*)
Coal 1	2.01	1280	0.30	12	0.28	1.4
Coal 2	5.24	1319	1.73	23.79	0.31	2.38
Coal 3	9.56	1420	2.31	34.26	0.15	3.8
Gangue 1	3.26	2460	1.19	30	0.24	3.5
Gangue 2	12.10	2630	3.76	42	0.23	5.1
Gangue 3	18.30	2610	5.24	52	0.21	6.8
Gangue 4	21.50	2600	7.17	64	0.19	7.4

**Table 3:** Valid simulation records and physical-condition groups for coal–rock cutting.

Cutting State	Effective Coal Firmness Coefficient *f_m_*	Effective Gangue Firmness Coefficient *f_y_*	Effective Volume Ratio	Valid Records	Physical-Condition Groups
All-coal	1.4, 2.38, 3.8	—	1:0	3	3
Single gangue layer	1.4, 2.38, 3.8	3.5, 5.1, 6.8, 7.4	1:1; 1:3; 3:1	35	35
Double gangue layers	1.4, 2.38, 3.8	(3.5, 5.1), (3.5, 6.8), (3.5, 7.4) (5.1, 6.8), (5.1, 7.4), (6.8, 7.4)	3:1:1; 2:2:1; 2:1:2 1:2:2; 1:3:1; 1:1:3	108	108
All-rock	—	3.5, 5.1, 6.8, 7.4	0:1	12	4
Total	—	—	—	158	150

**Table 4:** Main parameters of the cutting motor.

Parameter	Unit	General Value	Stator Value	Rotor Value
Rated Power	kW	55	-	-
Rated Voltage	V	1140	-	-
Pole Pairs (N_p_)	-	2	-	-
Rated Speed	r/min	1471	-	-
Rated Frequency	Hz	50	-	-
Mutual Inductance	H	0.224	-	-
Resistance	Ω	-	0.4	0.589
Inductance	H	-	0.225	0.255
Moment of Inertia	kg·m^2^	-	-	1.662

**Table 5:** Statistical characteristics of vibration acceleration in three directions under a representative single hard-gangue-layer condition.

Cutting Condition	Direction	Maximum/(mm·s^−2^)	Minimum/(mm·s^−2^)	RMS Value/(mm·s^−2^)
Single gangue layer	X	16,884.0	−12,205.6	1973.3
	Y	23,082.5	−35,401.8	4384.0
	Z	22,434.5	−21,985.1	3287.5

**Table 6:** Dataset partition based on physical condition groups.

Cutting State	Training Groups	Training Windows	Validation Groups	Validation Windows	Test Groups	Test Windows
All-coal	1	100	1	100	1	100
Single gangue layer	25	2500	5	500	5	500
Double gangue layers	76	7600	16	1600	16	1600
All-rock	2	600	1	300	1	300
Total	104	10,800	23	2500	23	2500

**Table 7:** Main architectural parameters of CORRECT-Net.

Component	Main Settings
SincNet front end	64 Sinc filters per modality branch; kernel length = 129; minimum low cutoff frequency = 5 Hz; minimum bandwidth = 10 Hz; Hamming window
Local CNN	Output channels = 64 and 32; kernel sizes = 9 and 5
Transformer encoder	Model dimension = 96; 2 encoder layers; 4 attention heads; feed-forward dimension = 384; dropout = 0.20
Graph representation	Four semantic nodes: vibration-local, vibration-global, current-local, and current-global
GATv2 module	4 attention heads; hidden dimension = 64 per head
Reliability-estimation branch	MLP structure = 128–64–1
Graph fusion	Fused representation dimension = 256
Classifier	MLP structure = 256–128–4

**Table 8:** Sensitivity of recognition performance to window parameters.

Window Length/Points	Window-Start Interval/Points	Accuracy/%	Macro-F1/%	Hazardous-Condition Miss Rate/%
800	75	92.71	90.43	0.91
1200	75	94.52	93.06	0.86
1600	75	96.20	95.14	0.58
2000	75	95.90	94.85	0.71
1600	150	95.86	94.68	0.77

**Table 9:** Classification performance of CORRECT-Net on the test set.

Class	Precision/%	Recall/%	F1-Score/%	Miss Rate/%	False-Alarm Rate/%
All-coal	87.77 ± 2.44	100.00 ± 0.00	93.47 ± 1.38	0.00 ± 0.00	0.58 ± 0.13
Single gangue	91.18 ± 0.52	93.00 ± 0.00	92.08 ± 0.27	7.00 ± 0.00	2.25 ± 0.15
Double gangue	98.65 ± 0.20	96.25 ± 0.17	97.44 ± 0.18	3.75 ± 0.17	2.33 ± 0.35
All-rock	95.25 ± 0.96	100.00 ± 0.00	97.56 ± 0.50	0.00 ± 0.00	0.68 ± 0.14
Macro-average	93.21 ± 0.38	97.31 ± 0.04	95.14 ± 0.21	—	—

**Table 10:** Progressive ablation results of CORRECT-Net.

Model	SincNet	Transformer	ResGATv2	Accuracy/%	Macro-F1/%	Hazardous-Condition Miss Rate/%
M-Base	—	—	—	87.40 ± 0.26	84.57 ± 0.34	1.46 ± 0.13
M-Sinc	√	—	—	91.28 ± 0.20	89.03 ± 0.18	1.04 ± 0.08
M-Trans	√	√	—	93.24 ± 0.17	91.57 ± 0.25	0.79 ± 0.07
CORRECT-Net	√	√	√	96.20 ± 0.11	95.14 ± 0.21	0.58 ± 0.13

**Table 11:** Recognition results of different models and input modalities.

Model	Modality	Accuracy/%	Macro-F1/%	Hazardous-Condition Miss Rate/%
SVM	Vibration	73.88 ± 0.43	65.81 ± 0.25	7.33 ± 0.13
1D-CNN	Vibration	86.04 ± 0.30	80.15 ± 0.24	3.17 ± 0.08
1D-CNN	Current	76.52 ± 0.31	68.65 ± 0.21	6.75 ± 0.12
1D-CNN	Vibration + Current	87.40 ± 0.26	84.57 ± 0.34	1.46 ± 0.13
Bi-LSTM	Vibration	91.96 ± 0.22	88.13 ± 0.29	1.88 ± 0.09
Bi-LSTM	Current	81.72 ± 0.24	74.47 ± 0.20	4.96 ± 0.08
Bi-LSTM	Vibration + Current	94.12 ± 0.15	91.53 ± 0.23	1.33 ± 0.16
TCN	Vibration	89.44 ± 0.23	84.32 ± 0.18	2.67 ± 0.08
TCN	Current	78.56 ± 0.32	71.06 ± 0.19	5.88 ± 0.07
TCN	Vibration + Current	92.20 ± 0.22	88.61 ± 0.36	1.75 ± 0.13
CORRECT-Net	Vibration	94.68 ± 0.15	92.33 ± 0.14	1.25 ± 0.13
CORRECT-Net	Current	89.32 ± 0.21	84.17 ± 0.22	2.54 ± 0.08
CORRECT-Net	Vibration + Current	96.20 ± 0.11	95.14 ± 0.21	0.58 ± 0.13

**Table 12:** Computational efficiency of CORRECT-Net and the baseline models.

Model	Parameters	FP32 Weight Storage/MiB	FLOPs/GFLOPs	Batch-1 Latency/ms
1D-CNN	78,052	0.30	0.065	0.99
TCN	120,708	0.46	0.329	3.58
Bi-LSTM	348,484	1.33	1.101	20.41
CORRECT-Net	1,031,111	3.93	0.195	7.91

**Table 13:** Robustness results of CORRECT-Net under different disturbance conditions.

Condition	Accuracy/%	Macro-F1/%	Hazardous-Condition Miss Rate/%
Clean	96.20 ± 0.11	95.14 ± 0.21	0.58 ± 0.13
Gaussian noise, σ = 0.01	95.68 ± 0.15	94.68 ± 0.43	0.54 ± 0.13
Gaussian noise, σ = 0.03	94.36 ± 0.21	92.90 ± 0.10	0.75 ± 0.09
Gaussian noise, σ = 0.05	92.76 ± 0.24	90.59 ± 0.13	1.13 ± 0.08
Vibration modality missing	86.56 ± 0.37	83.08 ± 0.34	3.46 ± 0.24
Current modality missing	92.44 ± 0.25	90.94 ± 0.46	1.33 ± 0.28
Random modality dropout, *p* = 0.10	93.00 ± 0.29	89.97 ± 0.14	1.54 ± 0.07
Amplitude shift, ±10%	93.48 ± 0.27	91.10 ± 0.42	1.25 ± 0.13

**Table 14:** Transfer recognition results under different experimental-data adaptation ratios.

Ratio	Adapt. Windows	Test Windows	Accuracy/%	Macro-P/%	Macro-R/%	Macro-F1/%	Hazardous-Condition Miss Rate/%
0%	0	60	91.33 ± 5.19	92.28 ± 5.08	91.33 ± 5.19	91.32 ± 5.26	3.11 ± 3.72
5%	12	60	93.67 ± 3.98	94.38 ± 3.45	93.67 ± 3.98	93.60 ± 4.09	1.78 ± 1.86
10%	24	60	95.00 ± 3.12	95.23 ± 2.98	95.00 ± 3.12	94.98 ± 3.13	1.78 ± 1.86
20%	48	60	96.33 ± 0.75	96.65 ± 0.71	96.33 ± 0.75	96.32 ± 0.75	0.00 ± 0.00
50%	120	60	98.67 ± 1.39	98.79 ± 1.24	98.67 ± 1.39	98.68 ± 1.38	0.00 ± 0.00

## Data Availability

The raw data supporting the conclusions of this article will be made available by the authors upon reasonable request to the corresponding author.
